# The Innovation and Development Path of Cultural and Creative Industries in Anhui Province, China: Nvivo12-Based Policy Text Analysis

**DOI:** 10.1155/2022/6202746

**Published:** 2022-07-20

**Authors:** Hui Zeng, Lei Yang

**Affiliations:** School of Art, Anhui University of Finance and Economics, Bengbu 233030, China

## Abstract

The policy planning of cultural and creative industries is the leading factor affecting the development of cultural and creative industries and is also an important factor in driving China's economic transformation. How to combine the policy with the actual industrial development and how to scientifically assess the construction effect of cultural and creative industries and build the corresponding planning system are the key points of the current development of cultural and creative industries in Anhui Province, China. The study first analyzes the regional cultural characteristics and development status of Anhui Province, then uses Nvivo12 qualitative software to conduct grounded research and qualitative text analysis on 20 Chinese cultural and creative industry development policy texts, and summarizes three core elements that promote the development of China's cultural and creative industry: “development task,” “development goal,” and “development guarantee.” Then, according to the three core elements of the text, we propose policy recommendations for the innovation and development of Anhui's cultural and creative industries: focus on “cultural heritage and sustainable development” policy formulation, explore Anhui's regional characteristics and culture; strengthen the development of urban-rural cultural integration and mechanism innovation; optimize and adjust the structure and supply of cultural industries; top-level design to clarify the strategic direction of culture, and coordinate the research tasks to empower industrial innovation. Relying on new media means, it integrates three major development strategies to build a new mechanism for internationalized communication of cultural and creative industries in Anhui Province, develop regional advantageous industries, form advantageous cultural industry clusters, effectively spread local culture, and promote sustainable development of local economy.

## 1. Introduction

With the arrival of the Internet era, social development begins to enter the era of digital information. Contemporary Chinese society is in a critical period of fusion and collision of various cultural and creative trends at home and abroad, and the attention to the development of cultural resources is gradually increasing. Regional cultural resources contain unique cultural genes and cultural values, and are important carriers of cultural creative design. Anhui Province's cultural resources are all-encompassing, and the distinctive and diverse regional culture is remarkable. The cultural and creative product design with Anhui's regional cultural elements as the creative source not only meets today's demand for design quality, but also is a physical witness to inherit the regional culture and maintain the national spirit. Therefore, while the regional economy is developing, how to integrate regional culture with cultural and creative product design and improve the bottlenecks and drawbacks of creative industry development according to local conditions has become an urgent problem to be solved. Cultural and creative industries are the epitome of Anhui Province's regional culture, and the development of cultural and creative industries is of great significance to the improvement of Anhui Province's comprehensive industrial strength. Relying on the new opportunities of Anhui River Cultural Industry Belt, South Anhui International Cultural Tourism Demonstration Zone, and the construction of regional industrial clusters in North Anhui, strengthening cultural exchanges with the world, creating cultural industry integration mechanism, and building cultural industry brands with Anhui Province's regional characteristics are effective ways to spread the connotation of Anhui Province's regional culture and inheritance development. Based on the grounded research method and combined with qualitative text analysis method, this paper provides an objective description and analysis of the research on Anhui regional economic development and cultural and creative products based on the content of text analysis by combing the policies related to China's cultural and creative industries in the new era, extracting conclusions from the policy text analysis, and providing a new perspective for the research on the innovation and development of special cultural and creative products in Anhui Province.

## 2. Related Research

### 2.1. Regional Cultural Characteristics of Anhui Province

Regional culture is a cultural tradition that has been formed by the people in a certain area during their long-term production and life, has been passed down from generation to generation, and is regional, developmental, and historical in nature. Anhui Province, as one of the birthplaces of early mankind in China, has a long history and a profound regional traditional cultural heritage, with a wide variety and distinctive features, and is an important part of Chinese civilization.

Geographically, Anhui Province is located in the east of China, with a vast area, in the middle and lower reaches of Yangtze River and Huaihe River, crossing the north and south and connecting the east and west. Under the influence of the unique regional environment and humanistic conditions, Huaihe culture, Anhui River culture, and Huizhou culture, which is a culture of humanity, literature, thrift, and righteousness, have been born, covering various aspects of folk customs, historical traditions, lifestyles, literature and arts, and each with its own characteristics [1]. Huaihe culture is concentrated in the Huaihe River basin area, which combines the characteristics of Jingchu culture, Central Plains culture, and Wu-Yue culture, and is compatible and open, containing cultural contents such as Confucianism of Confucius and Mencius, Jian'an literature, historical and cultural relics such as the ancient Shou County City and Tianyang Tianjing Palace, as well as intangible cultural heritage such as flower drum dances and Huabei clapper plays. The culture of Wanjiang River is located in the central basin of Yangtze River, connecting the north and the south through the waters of Yangtze River, and the interpenetration of different regional cultures has formed the unique landscape culture of Wanjiang River, with diversity and uniqueness. Huizhou culture, as the most representative culture in Anhui Province, is mainly distributed in the Xin'an River basin in the mountainous area of southern Anhui, and the ancient Huizhou prefecture, with six counties, carries the essence of Huizhou culture from ancient city buildings, cultural neighborhoods to famous scenery, and historical allusions, while folk arts such as Nuo dance, Huizhou opera, Wenfang Sijiao, and Xin'an literature and arts also blossom and complement each other.

With the development of the times and the leap of history, the regional culture of Anhui Province has gone through germination, development, inheritance, and internalization, and with its superior geographical location and rich cultural resources, it has formed the distinctive regional culture of Anhui Province [2]. The long history and cultural deposits provide valuable resource advantages for art creation, while the unique regional environment and cultural elements provide rich sources of inspiration for the selection of topics, design ideas, and expressions for the research and development of cultural creative products.

### 2.2. The Current Situation of Cultural and Creative Products in Anhui Province

#### 2.2.1. Homogenization Dilemma Highlighted and Weak Brand Awareness

Cultural creativity is the essential feature that distinguishes cultural and creative products from other commodities. Regional culture is an important part of Chinese culture and is a landmark symbol that distinguishes it from other cultures. In the current environment where cultural and creative themes are prevalent, it seems that cultural and creative products all over the world have similar faces, but the simple transplantation of cultural elements often floats on the surface, lacking novelty and individuality [3]. At the same time, most enterprises have weak brand awareness of cultural and creative products, have not yet formed independent cultural and creative brands, and rely on third-party services to undertake them, with insufficient product originality and lack of systematization, which reduces their own value in terms of competitiveness, and it is difficult for cultural and creative products without brand attributes to impress and resonate with consumers, which is not conducive to the effective promotion of cultural and creative industries in Anhui Province and even nationwide.

#### 2.2.2. Unclear Feature Orientation and Insufficient Design Creativity

Regional characteristics are unique and unrepeatable, and are the manifest expression of cultural heritage. As one of the carriers of culture, the design and development of cultural and creative products need to fully grasp the characteristic cultural connotation and precisely locate the characteristic cultural elements to ensure artistic innovation as well as to accomplish practical realization [4]. At present, the design of cultural and creative products in Anhui Province is still in the development and exploration stage, lacking accurate positioning analysis, with a single form, monotonous visual image, unable to deeply explore the regional cultural resources and properly interpret the local culture, and lacking genetic resonance leading to the lack of culture and innovation.

Cultural creative products are aimed at innovation, creation, and creativity as the fundamental means, emphasizing the revitalization of traditional culture, satisfying the material and spiritual needs of the people, and realizing the sense of identity and belonging to culture. Most of the cultural and creative products in Anhui Province show programmed development, most of them use traditional material carriers and do not develop new products based on the people's needs, the functions are not conveyed carefully enough, the design creativity is not enough, they cannot naturally integrate the regional cultural meaning with the products effectively, they lack deep thinking about the design themes and creative sources, and it is difficult to arouse consumers' psychological resonance.

## 3. Research Design

### 3.1. Text Selection

“Cultural and creative industry policy” refers to a series of strategic plans or guidelines formulated by the state, government, or relevant ministries and organizations to accelerate the development of China's national economy and promote the transformation and upgrading of industrial lifelines. This paper follows the principles of authority and standardization, while taking into account the timeliness of policy research, sets the screening period from January 1, 2017, to December 31, 2021, takes the relevant policy documents promulgated by the Central Committee of the Communist Party of China, the State Council, the Ministry of Culture and Tourism, and various departments as the objects of analysis, selects policy documents closely related to the development of cultural enterprises and the upgrading policies of cultural and creative industries by manual screening methods, under the premise of ensuring accuracy, combining with the actual situation and reflecting realistic problems, and finally selects 20 valid policy documents (Table 1).

### 3.2. Research Methods

This paper adopts two research methods, the first of which is based on root theory, an approach to qualitative research proposed by American scholars Glaser and Strauss, which is a special methodology for building a theoretical framework from in-depth analysis of empirical data. This approach emphasizes the acquisition of raw data from actual observations, the induction and summarization of new concepts through a bottom-up approach, and the identification of intrinsic links between concepts, which can then be taken to the theoretical level, in order to identify and solve neglected and potential problems in the direction of the research and explore a broader research field [5]. Specifically, we first define the research problem, explore literature sources, collect and organize empirical data, codify the policy text in open, spindle, and selective levels, continuously adjust and improve the theory through saturation testing, and finally form a complete theoretical model. Secondly, the qualitative analysis software Nvivo 12.0 Plus is used to conduct qualitative text analysis on 20 cultural and creative industry development policies promulgated by various Chinese departments that have been collected and combine the policy requirements at each stage with China's development strategy, from which the basic contents of the policy changes of transformation and development of China's cultural and creative industry and enterprise upgrading since 2017–2021 are sorted out, so as to provide a reasonable reference and reference for Anhui Province's regional cultural and creative industry planning and development in Anhui Province. Qualitative text analysis, as a systematic analytical research method, places the research question at the core of the entire analysis process and emphasizes the key role played by the interpretation of the text and the interpretation of its inner meaning [6, 7].

The research tool for this paper is the Nvivo 12.0 Plus qualitative research software developed by QSR Australia. Nvivo software is mainly used for data analysis in qualitative research, which can help researchers analyze the collection, storage, examination, and processing of information from pictures, texts, tables, videos, audios and web pages, etc. [8]. After the collated policy text information is imported into the software, advanced data management control system, search statistics, and query visualization tools are used to initially interpret the policy content structure, dig deeper for more valuable messages, summarize the laws, and reasonably predict the future evolution trend of the research field, which can effectively enhance the credibility, rigor, and authenticity of the analysis of grounded theory research.

### 3.3. Research Process

#### 3.3.1. Text Word Frequency Characteristics

**Figure 1:**
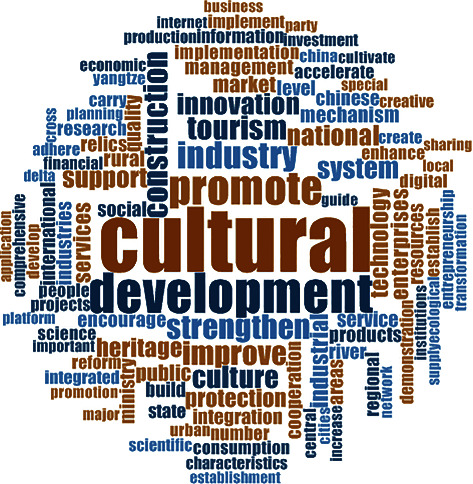
Policy document text word cloud.

The “word frequency” function in Nvivo shows the number of occurrences of all words in the policy text, and the “word cloud” function is an important visual representation of the word frequency, which can visualize the frequency of words in the policy text.  Figure 1 shows the word cloud of China's cultural and creative industry development policies from 2017 to 2021, the 100 words with the highest frequency in the policy text are selected, and the size of the words represents the number of frequency of words in the policy text; the larger the words are, the more they appear, and the smaller the words are, the less they appear. The “word cloud” reflects two distinctive features: the high-frequency words “Cultural, Innovation, System, National, Integration” indicate that the main issue to be addressed in China in the recent stage is policy reform and innovation. The terms “Industry, Tourism, Heritage, Public, Rural” reflect China's recent focus on upgrading its cultural and creative industries through the development of rural tourism and intangible cultural heritage. The “word cloud” function effectively identifies the characteristics of high-frequency words as an intuitive presentation, but the shortcoming is that it is difficult to judge the exact value of each word frequency.

To make up for the shortage and further analyze the characteristics of vocabulary usage in the policy documents for the development of China's cultural and creative industries in depth, the top 30 most frequently used terms are listed in Table 2. By categorizing the types of high-frequency words, we know that there are five main types of high-frequency words: first, the theme words: Cultural, Industry, National, Technology; second, the development objects: Tourism, Heritage, Public, Relics; third, the development subjects: System, Enterprises, Rural, Mechanism; fourth is the development task: Innovation, Support, Protection, Quality; fifth is the development verb: Promote, Strengthen, Improve, Encourage. The word frequency statistics table reflects the five most commonly used vocabulary categories in China's cultural and creative industry development policy documents from 2017 to 2021, systematically and visually demonstrating the word frequency usage characteristics of policy texts.

#### 3.3.2. Implementation of Data Coding

First of all, the policy texts of China's cultural and creative industry development with a total sample of 20 were imported into Nvivo 12.0 Plus software, new research projects were created, the bottom-up logical reasoning method of the grounded theory method was applied, and the imported texts were open coded, spindle coded, and selectively coded through descriptive language on the basis of careful reading of the policy text materials. The research sets up coding corresponding nodes according to the theme identification, establishes a theoretical research framework, and prepares more detailed coding by deeper mining of the nodes [9].

*(1) Open coding*. The open coding primary category is derived from imported policy text documents. In the process of open coding, the research always maintains open thinking and rigorous attitude, and uses Nvivo 12.0 Plus software to make preliminary coding of Chinese cultural and creative industry development policy texts, and by deeply sorting and grasping the contents of 20 policy texts, a total of 1,528 free nodes are obtained, and the free node class belongs to the primary stage in the subordination relationship, which is related to Chinese cultural creative industry development [10]. Immediately afterward, 1528 liberal nodes were analyzed for analogization, and nodes with the same concepts were merged and given new concepts to obtain 76 initial concepts, as shown in Table 3.

*(2) Spindle-type coding*. The purpose of spindle coding is to analyze in depth the various links that exist between concept groups and primary genera, and thus to rationally discover the organic links between the various parts of the policy text document material [11]. In order to make the coding more directional and accurate, the study sorted and summarized the 76 initial concepts obtained from the open coding, analyzed the internal connections among the concepts, and finally obtained 16 thematic genera. Thematic genus is obtained from qualitative analysis on the basis of the initial concept and belongs to the intermediate stage of subordination, which is also an intermediate factor related to the development of China's cultural and creative industries, that is, “Cultural Talent Team Development,” “Cultural Science and Technology Innovation Construction,” “Revitalizing Rural Cultural Industries,” “Diversification of Cultural Industry Integration,” “Regional Cultural Support and Innovation,” “Cultural Tourism Service System Construction,” “Cultural Market System Construction Goals,” “Cultural Services Shared Goals,” “Urban and Rural Cultural Integration Development Goals,” “Objectives of Institutional Reform of Cultural and Creative Industries,” “Cultural Heritage and Sustainable Development Goals,” “Institutional Guarantee,” “Policy Environment Guarantee,” “Leadership Organizational Guarantee,” “Economic Coordination Guarantee,” and “Public Service Guarantee.”

*(3) Selective coding*. In order to identify the core genera of the study, based on the inductive analysis of the 16 thematic genera obtained from the main axis coding, the specific characteristics and connections among the thematic genera were further explored in conjunction with other valid information in the research policy text materials, and the inclusiveness and relevance of the concepts were sought [12]. Core genus is the result of qualitative research analysis of thematic genus, which belongs to the advanced stage of subordination and is a macro factor related to the development of China's cultural and creative industries. The research builds a solid theoretical research system by analyzing, sorting, and integrating the three-level coding of the policy text and the coding content, and forms three core elements of the development strategy of China's cultural and creative industries: “development task,” “development goal,” and “development guarantee,” as shown in Table 4.

## 4. Research Results and Analysis

**Figure 2:**
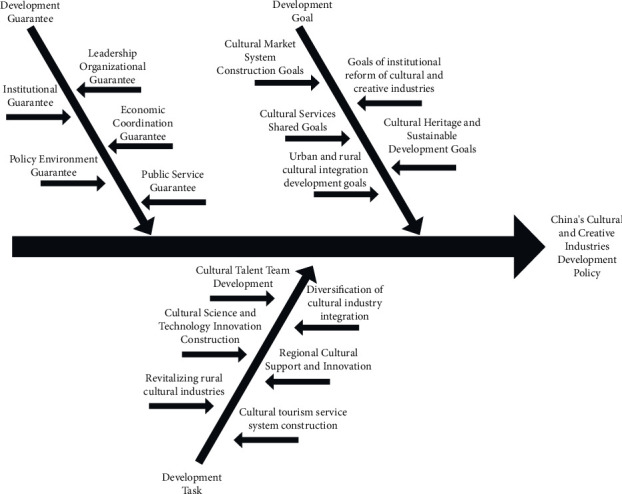
Structural model for the development of China's cultural and creative industries.

This study inductively constructs a development strategy system for China's cultural and creative industries based on 20 selected Chinese cultural and creative industry development policy texts, conducts qualitative textual research and analysis on the policy text data, proves the applicability of the grounded theory research method to the study of cultural and creative industry development, and summarizes the basic framework of China's cultural and creative industry development strategy on this basis, and the three core factors of “development task,” “development goal,” and “development guarantee” are derived; see Figure 2. The “development task” is an important element in the formation of China's national governance system and an important support for the development of a harmonious socialist society. The task emphasizes strengthening the excavation and dissemination of the excellent regional culture of the Chinese nation, adapting traditional regional culture to contemporary culture, coordinating with contemporary social development, enhancing the country's cultural soft power, and promoting a distinctive cultural spirit with contemporary values. The “development goal” aims at building a socialist cultural power and achieving cultural reform and development, emphasizing accelerating the development of cultural industries, promoting the transformation of distinctive cultural industries into pillar industries of China's economic development, building a modern cultural industry system, and improving the cultural market mechanism to provide theoretical support for the realization of the tasks. The “development guarantee” means that the Chinese government takes various security measures, accumulates and summarizes experience in cultural development management, and provides strong power guarantee, correct value guidance, and extensive intellectual support for the development of cultural and creative industries with Chinese characteristics. Specific explanation is given below.

### 4.1. Development Task and Development Goal Are Highly Compatible

The qualitative textual analysis revealed a high degree of correlation between the coding of “development task” and “development goal,” showing a correspondence and support relationship. On the one hand, it reflects the rigor of the textual content and logical arrangement of China's cultural and creative industry development policies, and on the other hand, it expresses the two-way linkage between “development task” and “development goal” [13]. For example, the first-level sub-node “regional cultural support and innovation” (development task) contains 226 reference points such as “building a regional development platform,” “fostering a regional entrepreneurial cluster,” and “innovating a regional cultural operation mechanism,” which together depict the framework of “cultural heritage and sustainable development goals” (development goal), providing a concrete path for the construction goals of cultural industries through task display and work allocation, and the corresponding “development goal” also indicates the direction for the planning and construction of “development task.”

The “development task” aims to achieve the “development goal,” and through rational value analysis and measurement, it plans strategic contents that meet social and spiritual values, providing theoretical guarantee for the construction and development of cultural and creative industries, as well as reasonable, scientific, and safe practical measures for the development of cultural and creative industries, highlighting the scientific and cutting-edge vision of the development strategy text of Chinese cultural and creative industries and the cause of Chinese cultural and creative industries, and providing favorable reference for regional culture comparison, study, and reference.

### 4.2. Regional Cultural Support Innovation Is the Focus of Development

Regional cultural support and innovation construction is not only an important factor in sustaining the development of China's cultural and creative industries, but also a powerful guarantee of inheriting Chinese culture and a characteristic symbol of mapping China's cultural soft power [14]. The regional culture of Anhui Province is still facing realistic resistance such as cultural nationalism and lack of policy support. Due to the paucity of research time and the limitation of sample space, the regional culture of Anhui Province has not been deeply excavated, and the focus has not yet shifted to the direction of combining the development of regional culture with contemporary development. The statistical table of policy text codes shows that the first-level sub-node “regional cultural support and innovation” is the code under the tree node “development tasks,” and the number of reference points is the largest year-on-year, accounting for 14.8%. In the text of China's cultural and creative industry development plan, there are more codes describing regional cultural support and innovation of the first-level sub-node, such as “optimize the structure and layout of regional cultural industry,” “strengthen regional cultural science and technology support,” “inherit and revitalize regional national culture,” and “innovation of regional cultural industry transformation.”

Regional culture is the foundation and accumulation of cultural work such as cultural service, cultural innovation, cultural development, and cultural transformation [15]. With the development of the times and policy changes, China's focus on the construction of cultural and creative industries is constantly changing, and the people's cognition and demand for regional culture also tend to be knowledge-based, which in turn influences the regional cultural industry to carry out policy innovation and development transformation around characteristic regional culture and resources, devotes itself to ensuring the high quality and diversified content, and forms of the cultural and creative industry construction system, so as to build a characteristic cultural industry model and tell the Chinese cultural story while providing reference and reference for other industrial development in China. It is the theoretical perspective of cultural development to solve cultural problems from the standpoint of cultural innovation and policy protection, to grasp the rationality and legality of policy protection, to strengthen the protection of intellectual property rights of cultural and creative industries on the one hand, to explore local regulations for Anhui Province, and to actively implement the development mode of digital copyright protection on the other hand, to distinguish the intellectual property properties of cultural and creative industries, to make a clear definition of the characteristics of cultural and creative products, and to implement a hierarchical protection system for cultural resources with special characteristics of Anhui Province. On the other hand, we should distinguish the intellectual properties of cultural and creative industries, make clear definition of characteristics for cultural and creative products, implement graded protection system for cultural resources with special characteristics in Anhui Province, effectively improve the accountability mechanism of Anhui government, and guide the rapid and stable development of cultural and creative industries in Anhui Province through the power of comprehensive legal system.

### 4.3. The Policy Guarantee That the Allocation of Attention Is Slightly Inadequate

By analyzing the text coding statistics table of China's cultural and creative industry development plan, we know that there are 215 reference nodes of the tree node “development guarantee,” accounting for 14.07% of all reference points, which proves that this part accounts for less space in all the text sample data, and there is a practical dilemma that the allocation of attention to policy guarantee is slightly insufficient. In the sub-node of institutional mechanism guarantee, the corresponding code expressions are mostly “establish and improve the management system and operation mechanism of cultural industry,” “promote cultural enterprises to establish and improve the modern enterprise system with cultural characteristics, and improve the comprehensive assessment and evaluation index system of social and economic benefits,” etc. [16]. These expressions all have some contents that need to be refined, such as how to coordinate the planning, management, and operation of the system and mechanism of cultural industry, how to ensure a balanced relationship between the systems, and how to implement the allocation of resources and acquisition of benefits among cultural enterprises, which is not conducive to the completion of cultural development tasks [17, 18].

Although the issue of policy guarantee is pointed out to facilitate the development of cultural and creative industries, whether it can be implemented in practice needs attention, and the provision of specific criteria and reference indicators is conducive to the accurate assessment of the strategic objectives of cultural development. Therefore, after releasing the text of cultural and creative industry planning, China needs to carry out subsequent improvement work one after another, establish effective and reliable standard guidelines, provide decision-making and support for promoting the development of cultural and creative industries with high efficiency, and promote the comprehensive and coordinated development of cultural and creative undertakings.

## 5. Research Recommendation

Looking at the policy documents related to the development plan of China's cultural and creative industries in the past five years, the development requirements of cultural and creative industries are gradually standardized, the practice programs tend to be standardized, the research and development contents of cultural and creative industries are gradually specified and clarified, and the policy system of cultural and creative industries is basically established. Under the guidance of these policy texts, the development work of China's cultural and creative industries can effectively implement the practice of turning, the cultural industry talent team continues to grow, and the industrial innovation and creative means are increasingly enriched, effectively guaranteeing the sustainable development of cultural and creative industries. By drawing on the development policies of China's cultural and creative industries, this paper takes Anhui Province, which is rich in regional resources, as the research object, analyzes the main contents of the policy system, and explores the realistic basis for promoting the innovation and development path of Anhui Province's special cultural and creative industries with the purpose of pulling the R&D work of Anhui Province's regional cultural and creative industry resources. In view of this, the study puts forward the following suggestions to give full play to the regional advantages, make up for the shortcomings of development, and strengthen the weaknesses of the industry, so that the development of cultural and creative industries can be promoted solidly in the territory of Anhui Province.

### 5.1. Focus on “Cultural Inheritance and Sustainable Development” Policy Development Ideas, Digging Anhui Regional Characteristics of Culture

By analyzing the policy text, it can be found that the development and construction of China's cultural and creative industries are showing a changing trend from focusing on traditional cultural industry to focusing on cultural heritage and sustainable development [19]. The past vigorous development and construction focusing on traditional cultural industry has led to prominent environmental problems and irreversible consumption of resources and is facing the heavy responsibility of changing the development mode of cultural industry. At present, Anhui Province has rich cultural resources with profound and obvious characteristics, and the excavation of Anhui Province's regional characteristics is not only an effective measure to promote China's excellent regional culture, but also a representative to show the distinctive features of Chinese civilization. It is an important issue facing the development of China's cultural and creative industries to ensure the effective development of Anhui Province's regional cultural resources, focus on supporting the development of cultural and creative industries, and formulate effective policies.

Regional economic prosperity is the primary prerequisite for regional cultural development, which requires a reasonable and strict policy guarantee when addressing the development dilemma of cultural industries [20]. The local government of Anhui Province should focus on the policy of “cultural heritage and sustainable development” to formulate and improve specific R&D strategies and initiatives for cultural and creative industries, improve the organizational structure, coordinate the collaborative work among various units and organizations, and complete the refinement of the development of cultural and creative industries in theory and practice. Under the way of nodal and fragmented integration of regional culture excavation, the nodal objectives and development tasks are gradually accomplished, so that the development of cultural and creative industries in Anhui Province tends to be systematic and complete, and the heritage and sustainable development of Anhui Province's regional culture are effectively guaranteed.

### 5.2. Strengthen the Integrated Development of Urban and Rural Culture and Mechanism Innovation, and Optimize and Adjust the Structure and Supply of Cultural Industry

Culture is the foundation of the countryside, and cultural development drives the revitalization of the countryside. For a long time, the development of urban and rural culture within the territory of Anhui Province has been extremely unbalanced, and the level of rural cultural development has yet to be improved [21]. From the perspective of urban-rural integration development, rural cultural revitalization should reasonably draw on the development paths and experiences of urban cultural industries, start from the perspectives of folk culture, red culture, and intangible culture, tap the local market elements of cultural and creative industries according to local conditions, enhance the adaptability of rural cultural industries to the information era, reduce the conflict between urban and rural cultural development, strengthen urban-rural cultural integration development and mechanism innovation, better meet the growing material and spiritual cultural needs of rural people, improve the rural environment, develop the rural economy, and promote the overall revitalization of rural culture.

The development of regional cultural and creative industries in Anhui Province should not only grasp the geographical and cultural advantages and develop its own competitiveness, but also strengthen the penetration and radiation of regional cultural dissemination [22]. In the process of development, the government should play a leading and exemplary role, change the research concept and perspective, innovate the development strategy, consider the way of transformation and upgrading of cultural contents and expressions from the perspective of consumers, effectively combine the regional culture with science and technology, innovate the expressions of cultural creative products through information and Internet methods, optimize and adjust the structure of cultural industry and cultural supply, further activate the cultural market at home and abroad in Anhui Province, and establish the cultural brand and cultural industry with the image of Anhui Province's characteristics.

### 5.3. Top-Level Design Clarifies the Strategic Direction of Culture and Coordinates Research Task Arrangements to Empower Industry Innovation

China has the research experience of top-level design through planning over the years [23]. As for the top-level design of cultural and creative industry innovation and development in Anhui Province, the government and organizational units not only associate it with the overall cultural planning and building a strong country of cultural and creative industry, but also need to embed it in the visionary goal of cultural industry development, highlighting the direction and focus of cultural and creative industry development in Anhui Province. Specifically, the outline of the Yangtze River Delta Regional Integrated Development Plan promulgated by the State Council of China in 2019 clearly specifies the creation of a cultural and creative industrial park in Anhui Province by taking advantage of its distinctive features and good resources, and plans the strategic tasks and future direction of the development of cultural and creative industries in Anhui Province in the coming period of time, which is the right point for top-level design and collaborative planning by the Chinese government.

At the same time, the development and planning of cultural and creative industries in Anhui Province is a systematic project, which needs to arrange and allocate research tasks by taking into account and coordinating the governance of various industrial issues [24]. At the macro level, there are development and cultural differences between the north and south of Anhui Province, and the Anhui River culture, Huaihe culture, and Huizhou culture coordinate with each other to absorb and learn from each other's experience in the development of cultural and creative industries; in addition, it is necessary to bring into play the cultural resources and economic advantages of the southern region of Anhui Province to support the development of cultural and creative industries in the northern region of Anhui Province. At the meso-level, Anhui's cultural and creative industry development also involves regional cities and villages at different stages of development, so it is necessary to coordinate the relationship between these regions and properly arrange the tasks of cultural and creative industry development. From the micro-level, the development of cultural and creative industries in Anhui Province needs to be implemented to researchers, deal with the relationship between research experts and talent team training, and implement cultural talent team training and public service guarantee.

## 6. Conclusion

Overall, based on the strategic objectives of innovation and development, it clarifies the thematic changes and planning characteristics of China's cultural and creative industry development policies, and then summarizes and reflects on the experience of policy documents, which not only points out the research direction for the development of regional cultural and creative industries in Anhui Province, but also provides fundamental guidelines for the development of cultural and creative industries in other regions of China. Wu pointed out that “the development of cultural and creative industries nowadays begins to enter the era of brand strategy, and the construction of cultural and creative industries still has much room for improvement [25]. By studying the development of cultural and creative industries with regional characteristics in Anhui, it is proposed to establish and improve the development system and brand development working mechanism of cultural and creative industries with characteristics in Anhui Province, effectively and scientifically analyze the supply and demand of cultural and creative industry market and industrial chain, actively promote the innovation and brand construction of cultural and creative industries, create the mechanism of cultural new products and service platform, and give full play to the cultural advantages of Anhui Province with regional characteristics in order to expand international influence of Anhui Province culture and sustainable economic development.” It is expected that in the near future, the development of cultural and creative industries in Anhui Province will usher in qualitative changes, adapt to the needs of the development of the new era, and be at the forefront of cultural industry development.

## Figures and Tables

**Table 1:** Research access to policy texts.

Serial no.	Policy text name	Issuing department	Issue date
1	Cultural Industry Development Plan for “The 13th Five-Year Plan” Period of the Ministry of Culture	Ministry of Culture and Tourism	2017.4
2	Outline of the National Cultural Development and Reform Plan for “The 13th Five-Year Plan” Period	China State Council	2017.5
3	Opinions on Promoting High-Quality Development of Innovation and Entrepreneurship to Create an Upgraded Version of “Dual Innovation”	China State Council	2018.9
4	Interim Measures for the Management of China's Industrial Heritage	Ministry of Industry and Information Technology	2018.11
5	Further support the development of cultural enterprises, two provisions of the notice	China State Council	2018.12
6	Guidance on fostering the development of modern urban areas	China National Development and Reform Commission	2019.2
7	Guidance on promoting the revitalization of rural industries	China State Council	2019.6
8	Opinions of the General Office of the State Council of China on Further Stimulating the Consumption Potential of Culture and Tourism	China State Council	2019.8
9	Guidance on promoting the deep integration of culture and science and technology	Ministry of Science and Technology	2019.8
10	Cultural Industry Promotion Law of the People's Republic of China (draft for review)	Ministry of Justice	2019.12
11	Outline of the Yangtze River Delta Regional Integrated Development Plan	China State Council	2019.12
12	Opinions on promoting the high-quality development of digital culture industry	Ministry of Culture and Tourism	2020.11
13	Opinions on the promotion of high-quality development of public cultural services	Ministry of Culture and Tourism	2021.3
14	“The 14th Five-Year Plan” Cultural and Tourism Development Plan	Ministry of Culture and Tourism	2021.4
15	Opinions on further increasing development finance to support the high-quality development of the cultural industry and tourism industry	Ministry of Culture and Tourism	2021.4
16	“The 14th Five-Year Plan” Cultural Industry Development Plan	Ministry of Culture and Tourism	2021.5
17	Promote the development of industrial culture implementation program (2021–2025)	Ministry of Industry and Information Technology	2021.6
18	Opinions on promoting high-quality development of the central region in the new era	China State Council	2021.7
19	Several measures to further promote the development of cultural creative products of cultural heritage units	Ministry of Culture and Tourism	2021.9
20	“The 14th Five-Year Plan” Heritage Conservation and Science and Technology Innovation Planning	China State Council	2021.11

**Table 2:** Policy document text word frequency statistics table.

High-frequency keywords	Frequency	Weighted percentage (%)	High-frequency keywords	Frequency	Weighted percentage (%)
Cultural	2811	3.48	Industrial	457	0.57
Development	1591	1.97	Protection	457	0.57
Promote	1264	1.57	Enterprises	444	0.55
Industry	911	1.13	Services	434	0.54
Strengthen	772	0.96	Market	378	0.47
Construction	769	0.95	Public	377	0.47
Tourism	754	0.93	Rural	348	0.43
Improve	747	0.93	Mechanism	345	0.43
Culture	677	0.84	Quality	325	0.40
Innovation	635	0.79	Encourage	324	0.40
System	617	0.76	Management	320	0.40
National	599	0.74	Products	313	0.39
Support	574	0.71	Relics	312	0.39
Heritage	501	0.62	Integration	304	0.38
Technology	468	0.58	Cooperation	302	0.37

**Table 3:** Primary genera and initial concepts formed by open coding (part).

Initial concept	Primary genus
Development of new cultural industries	In line with the development trend of digital industrialization and industrial digitization, we will deeply apply 5G, big data, cloud computing, artificial intelligence, ultra-high definition, Internet of Things, virtual reality, augmented reality, and other technologies to promote the high-quality development of digital culture industry, and cultivate and grow new cultural industries such as online performance, digital creativity, digital art, digital entertainment, and immersive experience

Development of rural special culture	Vigorously develop county and rural special cultural industries, promote the integrated development of urban and rural areas, promote the flow of factors more to the countryside, build a number of cultural industry characteristics of the township and cultural industry characteristics of the village, promote rural characteristics of cultural resources, traditional craftsmanship and creative design, modern technology, the combination of the elements of the times

Promoting the digitization of cultural resources	The digital transformation and development of cultural resources allows outstanding cultural resources to “come alive” with the help of digital technology, combining the value content contained with the new form and elements of digital technology to achieve creative transformation and innovative development

**Table 4:** Policy text code statistics table.

Selective coding	Spindle-type coding	Reference points	Coverage (%)	Total (%)
Development task (737)	Cultural talent team development	66	4.31	48.23
	Cultural science and technology innovation construction	71	4.64	
	Revitalizing rural cultural industries	157	10.3	
	Diversification of cultural industry integration	86	5.62	
	Regional cultural support and innovation	226	14.8	
	Cultural tourism service system construction	131	8.56	

Development goal (576)	Cultural market system construction goals	92	6.02	37.70
	Cultural services shared goals	68	4.45	
	Urban and rural cultural integration development goals	184	12.04	
	Objectives of institutional reform of cultural and creative industries	53	3.48	
	Cultural heritage and sustainable development goals	179	11.71	

Development guarantee (215)	Institutional guarantee	45	2.95	14.07
	Policy environment guarantee	51	3.34	
	Leadership organizational guarantee	30	1.96	
	Economic coordination guarantee	27	1.76	
	Public service guarantee	62	4.06	

## Data Availability

The data used to support the findings of this study are available from the corresponding author upon request.
